# Regulation of Subcellular Protein Synthesis for Restoring Neural Connectivity

**DOI:** 10.3390/ijms26157283

**Published:** 2025-07-28

**Authors:** Jeffery L. Twiss, Courtney N. Buchanan

**Affiliations:** 1 Department of Biological Sciences, University of South Carolina, Columbia, SC 29208, USA; courtney.buchanan@uscmed.sc.edu; 2 Carolina Autism and Neurodevelopment Research Center, McCausland College of Arts and Sciences, University of South Carolina, Columbia, SC 20208, USA

**Keywords:** RNA transport, protein synthesis, RNA stability RNA-binding protein, integrated stress response

## Abstract

Neuronal proteins synthesized locally in axons and dendrites contribute to growth, plasticity, survival, and retrograde signaling underlying these cellular processes. Advances in molecular tools to profile localized mRNAs, along with single-molecule detection approaches for RNAs and proteins, have significantly expanded our understanding of the diverse proteins produced in subcellular compartments. These investigations have also uncovered key molecular mechanisms that regulate mRNA transport, storage, stability, and translation within neurons. The long distances that axons extend render their processes vulnerable, especially when injury necessitates regeneration to restore connectivity. Localized mRNA translation in axons helps initiate and sustain axon regeneration in the peripheral nervous system and promotes axon growth in the central nervous system. Recent and ongoing studies suggest that axonal RNA transport, storage, and stability mechanisms represent promising targets for enhancing regenerative capacity. Here, we summarize critical post-transcriptional regulatory mechanisms, emphasizing translation in the axonal compartment and highlighting potential strategies for the development of new regeneration-promoting therapeutics.

## 1. Introduction

Nervous system function requires connections between neurons and their innervation targets that are provided by long extensions of the neuronal cytoplasm known as dendrites and axons. Dendrites extend for up to 2 mm, receiving synaptic input and transmitting action potentials towards the cell body or soma. In contrast, axons often span much longer distances—over a meter or more in humans and other larger mammals—and typically transmit action potentials away from the soma towards a synapse. These distinct functions dictate that axons and dendrites contain unique pre-synaptic and post-synaptic functional domains composed of different proteins, respectively. Nevertheless, axons and dendrites share many of the same structural and signaling proteins that enable them to extend long distances and facilitate the directional transport of proteins, RNAs, and organelles. Though messenger RNA (mRNA) localization into dendrites initially gained wide acceptance in the neuroscience community [1], it later became clear that axons also contain mRNAs and synthesize proteins locally [2]. Indeed, the localization of mRNAs and translational machinery in axons and dendrites provide neurons with a renewable, ‘on-demand’ source of new proteins at distal sites, enabling rapid modification of their proteomes independent of protein transport from the soma. Since axons provide long-range communication in the brain, spinal cord, and peripheral nerves, they are particularly susceptible to disruption caused by traumatic injury. Consequently, axonal mRNA translation plays a crucial role in initiating and sustaining axon regeneration following injury [3].

The transport of RNAs to subcellular domains has been the focus of increasing interest over the past 20–30 years as advances in molecular and cellular biology tools have enabled high-specificity detection of nucleic acids and nucleic acid/protein complexes at single-molecule resolution. These technological advances have significantly enhanced our understanding of RNA localization dynamics. Long-range RNA transport relies on microtubule-dependent motor proteins (i.e., kinesin and dynein), while short-distance transport utilizes actin filament-dependent motor proteins (i.e., myosins). mRNAs, and/or non-coding RNAs associated with RNA-binding proteins (RBP) and motor proteins to form ribonucleoprotein (RNP) complexes. Adapter proteins mediate these interactions by either binding directly to the motor complex or indirectly to organelles such as endosomes, lysosomes, or mitochondria [4]. This emphasizes that RNPs are actively transported into axons via ATP-requiring motors rather than passive diffusion. Specificity and control of RNA localization and translation is regulated at multiple levels, including RNP assembly, RNP engagement with motor proteins, and the modulation of RBP/RNA interactions, including an RBP’s affinity for RNA binding [4]. These mechanisms contribute to translational regulation of mRNAs once they reach their locales in axons and dendrites and critically influence axon regeneration capacity and growth rates.

Here, we summarize the key mechanisms underlying post-translational regulation of mRNA localized in axonal compartments. We offer a perspective for how these mechanisms, governing axonal mRNA transport, stability, storage, and translation, influence axon growth and regenerative capacity. Finally, we discuss recent insights into how understanding these regulatory processes could inform new therapeutic strategies aimed at promoting neural repair and functional recovery following injury and disease.

## 2. Subcellular Synthesis of Neuronal Proteins

### 2.1. Functions of Locally Synthesized Proteins

Localized translation of neuronal proteins first came into acceptance for dendrites after early electron microscopy (EM) studies of the adult rat hippocampus identified polysomes at the base of dendritic spines [5]. This gave rise to the hypothesis that dendrite-synthesized proteins are utilized for synaptic plasticity by quickly responding to synaptic stimuli [6]. Experiments over the last 40 years have extensively validated this hypothesis, with particular acceleration in knowledge since the early 2000s. For example, the gene for the Activity-Related Cytoskeletal protein (ARC) is rapidly transcribed after synaptic activation and then its mRNA is transported into dendrites, with levels concentrating at activated synapses [7,8,9]. Locally synthesized ARC protein serves to increase neurotransmitter receptor endocytosis and promote synaptic plasticity [10]. Further oligomerization of CAMKII-phosphorylated ARC promotes memory consolidation [11] (Table 1).

Supporting the notion of a quick response to a stimulus, it was appealing to hypothesize that axons would similarly benefit from localized protein synthesis. Despite the much longer distances that axons traverse and some evidence for synthesis of proteins in axons from the mid-1960s to 1970s [12,13,14,15,16], the aforementioned EM studies of the adult rodent hippocampus did not detect polysomes in the pre-synaptic compartment [5]. However, high-resolution and high-sensitivity (non-isotopic) RNA detection methodologies aiming to visualize nascently synthesized proteins have shown that axons do in fact contain mRNAs, ribosomes, and other protein synthesis machinery, and they actively synthesize proteins. Importantly, axonal mRNA localization and protein synthesis have been documented during development and into adulthood [17,18,19,20]. In contrast to dendrites, axonally synthesized proteins seem to fulfill broader roles, including directional and regenerative growth, injury responses, retrograde signaling, and pre-synaptic plasticity [21]. Based on mRNAs known to localize into dendrites (e.g., prenylated and palmitoylated *Cdc42* mRNA isoforms [22]), the focus on synaptic plasticity proteins may underestimate the full extent of functions for dendrite-synthesized proteins.

RNA profiling studies have remarkably expanded our understanding of the axonal transcriptome, revealing that mRNAs can be remarkably enriched in axons compared to the soma in vivo [23]. The extended lengths of axons and their relative isolation from other neuronal components have provided more technical ease when isolating axonal contents as compared to dendrites. However, it is likely that the dendritic transcriptome is equally as complex as that of axons based on RNA sequencing studies of dendritic fields, where non-neuronal RNAs can be bioinformatically subtracted to yield a dendrite-enriched transcriptome [24]. Some interesting points come from evaluating axonal transcriptomes as well as from approaches used to isolate translationally active mRNAs from axons (i.e., the ‘axonal translatome’). First, there are many mRNAs encoding secreted and transmembrane proteins represented (for examples, see [25,26,27,28,29]). Consistent with this, several studies have shown components of the endoplasmic reticulum (ER) and Golgi apparatus in axons, though these organelles may not have the classic EM appearance that they do in the soma [30,31]. There are also a number of axon-synthesized proteins whose mRNAs are also translated in the soma and transported into axons, resulting in dual sources of protein. Since most mRNAs are not enriched in axons [32], soma-to-axon protein transport remains a significantly larger source of proteins. Further, we know of differences in axonal transcriptomes between different neuron types [32], but further work needs to be carried out to fully understand how axons of different neuron types utilize locally synthesized proteins. For example, actin isoforms (β-Actin and γ-Actin) are differentially localized, with *Actg* mRNA restricted to the soma in cortical and dorsal root ganglion (DRG) neurons but *Actg* mRNA localizing to motor axons. Locally synthesized β-Actin promotes axon branching in DRG neurons, while locally synthesized γ-Actin plays this role in motor neurons [33,34].

**Table 1:** Example functions of locally synthesized neuronal proteins.

Function	Locally Synthesized Neuronal Proteins	Description
Synaptic plasticity in dendrites	ARC, CaMKIIα, PDS-95	Allows for rapid modification of synaptic strength and plasticity, critical for memory formation and consolidation [7,8,9,10,11,35,36].
Dendrite growth	Palmitoyl- and Prenyl-CDC42 isoforms	Promotes dendrite growth and spine maturation (Palm-CDC42) and branching (Prenyl-CDC42) [22,37]
Axonal growth and guidance	β-Actin, γ-Actin, Cofilin, RhoA, GAP43, Prenyl-CDC42 isoform, HMGB1, NRN1	Drive growth cone dynamics, regulate axonal branching, and guide directional axon growth by enabling rapid, spatially restricted synthesis of cytoskeletal and signaling proteins [22,34,38,39]
Axonal injury response and retrograde signaling	Importin B1 (Kpnb1), RanBP1, Vimentin, mTOR, CALR, CREB3	Rapidly synthesized after injury to initiate local regeneration processes, growth cone formation, retrograde signaling, and stress responses critical for neuronal survival and recovery [20,40,41,42,43].
Axon survival	Bclw	Loss of *Bclw* mRNA transport into axons in neuropathy triggers axon degeneration [44,45]
Retrograde transport	LIS1	Modulates activity of retrograde motor protein dynein [46]
mRNA stability	KHSRP	RNA-binding proteins can regulate mRNA transport, stability, storage, and translation, enabling compartment-specific control of proteins [47]
Nuclear transcriptional regulation	CREB, LUMAN/CREB3, STAT3, ATF4, HMGN5	Allows for axonal synthesis of factors involved in nuclear transcriptional regulation with pro-growth or neurodegenerative outcomes [48,49,50,51,52].
Vesicle, protein, and membrane trafficking	BIP (GRP78), CALR, SNAP25, TC10	Facilitates local membrane repair, secretion pathways, and vesicle trafficking critical for axonal maintenance, growth, and injury responses [43,53,54,55,56,57]
Mitochondrial function and transport	Lamin B2, COXIV, COXVIIC, PINK1	Ensures local mitochondrial biogenesis, energy metabolism, and transport within axons [58,59,60]

Whether individual axon-synthesized proteins are functionally distinct from those derived via soma-to-axon protein transport is a fundamental cell biology issue that remains unresolved. It is appealing to hypothesize that locally synthesized proteins establish functional microdomains within axons that cannot be fulfilled by the soma-to-axon-transported protein. Consistent with this, Prenyl-CDC42 is synthesized in the soma and transported into axons, as well as made locally in axons. Growth promotion by CDC42 requires local synthesis and post-translational modification of Prenyl-CDC42, with the axon-synthesized protein localizing to the growth cone periphery while soma-synthesized Prenyl-CDC42 is restricted to the proximal growth cone [22]. Given the contributions of axonal protein synthesis to axon growth and functions, it will be critical to understand potential differences between axon-synthesized and soma-to-axon-transported proteins as we contemplate the potential impacts of any therapeutic strategies to alter axonal mRNA translation.

### 2.2. RNA–Protein Interactions Are a Key Determinant for Where and When Individual mRNAs Are Translated in Axons and Dendrites

Localization and translation of neuronal mRNAs is tightly regulated by RBPs, which recognize specific sequence or structural motifs within the mRNAs. Further, the RNA-binding proteins in axons and dendrites can be defined by the structures of the RNA-binding domains (e.g., K-homology [KH] domains, RNA recognition motifs [RRM], Arginine–Glycine-Rich [RG or RGG] motifs, etc.), as well as functionally by their bound mRNA populations. The vast majority of these proteins are ubiquitously expressed, so they also have functions in other cell types, and many have nuclear functions (e.g., RNA splicing). RNA–protein interactions form the basis for subcellular regulation of gene expression and are essential for enabling spatial and temporal control over protein synthesis in axons and dendrites. One of the most well-characterized examples is the localization of β-Actin (*Actb)* mRNA, which localizes in axons and dendrites through Zip Code Binding Protein 1 (ZBP1 also called IMP1 and IGF2BP1). ZBP1 binds a ‘zip-code’ motif in the mRNA’s 3′untranslated region (UTR) [61,62]. This binding actively inhibits *Actb* mRNA translation until the protein is tyrosine-phosphorylated by a Src family kinase; this phosphorylation event decreases ZBP1’s RNA-binding affinity, thereby releasing *Actb* mRNA for translation [63]. In general, 3′UTRs contain motifs for RNA localization and stability, while 5′UTRs contain motifs for translational regulation; however, this is not absolute and there are multiple examples for the impacts of 5′UTR and 3′UTR motifs on translational regulation and subcellular localization [39,64]. Interactions between 3′ and 5′UTRs can also impact translational regulation, and mRNAs can have multiple localization motifs [65]. Still, there are examples of motifs within mRNA’s protein coding sequence (CDS) and CDS influencing UTR-mediated localization [59,66,67]. At first glance, this makes for a very complicated system, but these different mechanisms provide a means to control not only where mRNAs go within the neuron but also when and where they are translated into new proteins.

Further, axonal mRNA interactions with the KH-type Splicing Regulatory Protein (KHSRP) can promote the decay of axonal mRNAs, thereby determining how much axonal protein is generated from a KHSRP target mRNA [47].

A striking feature of neuronal mRNA regulation is the apparent lack of shared primary sequence motifs among axon-localizing transcripts [4]. Despite considerable efforts, few universal sequence elements have been identified. However, recent high-throughput reporter assays have begun to identify candidate localization motifs across neuronal mRNAs. For example, AU-rich elements within 3′UTRs have been shown to facilitate localization through interactions with micro-RNAs and the RNA-binding protein HBS1L [68]. These findings suggest that secondary RNA structure, rather than linear sequence alone, plays a critical role in determining RBP recognition. Such structures, typically predicted as ‘stem’ and ‘loop’ formations based on intra-RNA Watson–Crick base-pairing, likely guide RNA binding. ‘Predicted’ is a notable descriptor here, and the reader should consider whether an identified structure has been proven to exist in vivo when evaluating reported motif structures. Beyond the secondary structure, tertiary RNA structures are formed through a variety of intramolecular interactions, likely also contributing to RBP motif recognitions, though the current methods used to identify or predict these structures are limited [69]. Motifs based on known RBP binding preferences have helped to identify localization elements in subsets of axonally localized mRNAs, including binding sites for SFPQ, HuD, and KHSRP that extend to other axonal mRNAs [45,70,71]. Interestingly, a primary sequence motif mediating the retention of mRNAs in the neuronal soma has been defined for PUM2, emphasizing that mRNA localization is not solely dependent on a transcript’s ability to enter axons or dendrites. Instead, some mRNAs are actively excluded from distal compartments via specific retention mechanisms [72].

Iterative functional testing of an mRNA sequence(s) for localizing activity has also proven to be a consistent means of identifying motifs by showing that an RNA segment is both necessary and sufficient for axonal localization. For UTRs, regions of high sequence conservation between species have some predictive value for identifying RNA regions to test for functional regulation [64,73]. For this approach, a putative RNA motif is inserted into a non-localizing reporter mRNA UTR and then expressed in cultured neurons to test for localizing activity through in situ hybridization for the reporter mRNA or based on changes in reporter protein levels [74]. Identified motifs can then be used for affinity isolation of RBPs, including for axons [75]. Studies with axoplasm preparations have shown that an RBP can bind to many different RNAs [76,77]. Gene ontology analyses of these bound mRNAs from the axoplasm of regenerating PNS axons point to shared functions for axon growth by proteins encoded by the bound mRNAs [77]. These findings suggest that axonally localizing RBPs have defined RNA regulons, a term initially applied for coordinating cellular responses through post-transcriptional regulation by RBPs [78]. For most of the RBPs in axons and dendrites, we know the full extent of the RNA regulons that they modulate in those structures.

### 2.3. Axonal mRNA Transcriptomes

Due to their exceptional length and relative anatomical isolation, methods used to isolate axons or alter their contents to relative purity have fueled more rapid discoveries for a range of axonally synthesized proteins compared to dendrites. RNA sequencing studies have indicated that a few thousand mRNAs can localize to axons—collectively termed the ‘axonal transcriptome’—with ribosome profiling revealing that many of these transcripts are actively translated in situ [23,25,26,27,28,29]. Though these transcriptomes and translatomes contain mRNAs encoding cytoplasmic, ER, mitochondrial, and secreted/transmembrane proteins, there is a surprising dearth of mRNAs encoding proteins with receptor or channel activities. It is conceivable that these membrane-linked proteins are preferentially synthesized in the soma and delivered to axons via fast axonal transport. Moreover, their activity can be closely modulated by ligand binding, potentially reducing the need for rapid ‘on-demand’ synthesis of these proteins in axons.

Interestingly, many axonally synthesized proteins are also synthesized in the soma and transported to axons as mature proteins or protein complexes, raising the possibility that the axon-synthesized version of a protein has unique functions or properties compared to the same protein delivered from the soma. We can see evidence of this with the Prenyl-CDC42 isoform, where the axon-synthesized version localizes to the growth cone periphery and supports axon growth, while its soma-synthesized version is restricted to the proximal growth cone [22]. Similarly, while β-Actin mRNA is one of the earliest identified axonally localized transcripts [18], studies estimate that over 90% of β-Actin protein in axons originates from the soma [17], and whether the site of synthesis influences β-Actin’s function remains unresolved. These findings underscore the need for more targeted studies to dissect the functions of axon- vs. soma-synthesized proteins.

While transcriptome profiling has significantly advanced, with next-generation sequencing providing genome-wide profiling for local transcriptomes, our ability to actively track mRNA transport in real time has lagged behind. For imaging, the single-molecule detection of mRNAs, including multiplex approaches, has enabled precise mapping of individual mRNAs along axons, including quantitative analysis of how levels of individual mRNAs respond to different stimuli. Further, a combination of single-molecule fluorescent in situ hybridization (smFISH) with immunofluorescence has been used to visualize colocalization of mRNAs and RBPs in both axons and dendrites [79]. Proximity ligation assays using high-specificity antibodies further allow for the visualization of RNA-RBP colocalization in a spatially resolved manner [80]. Additionally, spatial transcriptomics approaches have the potential to couple genome-wide RNA sequencing with microscopy [81], although their current limitations in Z-dimension resolution hinder the definitive identification of neuronal processes. While these methods have advanced our understanding of mRNA localization, they only provide static images, emphasizing the need to visualize the dynamics of mRNAs during their transit. Several methodologies have been developed for this, including tagging transfected cDNA constructs with motifs for bacteriophage RBPs like the MS2 and PP7 systems [82,83]. Although powerful, these tools often rely on transfection with overexpression, so the mRNA product of the transfected cDNA could differ in behavior from endogenous mRNAs. Still, these RNA-tracking approaches can be combined with the translation reporters outlined below to gain a more complete overview of an RNA’s subcellular fate [84]. Genetically modifying endogenous rodent alleles to include MS2 motifs has helped to overcome overexpression issues [85]; however, these models are labor-intensive and are not scalable for high-throughput applications. ‘Molecular beacons’, fluorophore-quenched fluorescent antisense oligonucleotides, have been used to visualize transported RNAs—when the beacon hybridizes to an RNA, the quencher loses fluorophore proximity and the beacon can be excited by fluorescent light [85]. These offer a complementary live imaging approach and have shown utility in tracking RNA localization, though they are underutilized in mammalian neural systems [76]. Emerging methods are poised to address these limitations. For example, CRISPR-based approaches, such as those developed by Ogawa et al. (2023), allow for the viral delivery of RNA-tagging constructs with improved scalability and reduced cost compared to the generation of new mouse lines [86]. Additionally, RNA aptamers that behave like fluorescent proteins (i.e., the initially developed ‘Spinach’) have potential for tagging endogenous alleles without the need for protein-based reporters [87,88]. Continued development of these approaches will be essential for dissecting the spatial and temporal dynamics of axonal mRNA localization and translation in neurons.

### 2.4. Ribonucleoprotein Complexes (RNPs) as Regulatory Platforms for RNA Transport, Stability, and Translational Regulation

Complexes of RNAs and RBPs in the cytoplasm, which we refer to here cumulatively as RNPs, have been described as granules based on their cellular functions as RNA transport granules, stress granules (SGs), and processing bodies (P-body) [89]. Though these structures are defined by their unique proteins or combinations of proteins, many of these granule-defining proteins can shuffle between the different granule types. It is intriguing to speculate that intermediate forms between these granule types exist or even that the transport granule, SG, and P-body define the vertices of a ternary RNP spectrum. A compelling example of this versatility is the core SG protein G3BP1, which also functions as a transport granule, localizing axonal mRNAs via hitchhiking on endo/lysosomes and binding to the Annexin A11 adapter protein [90]. SGs containing G3BP1 and other proteins are used to store unneeded, translationally silenced mRNAs during periods of stress. But axons utilize G3BP1 granules under non-stressed conditions to store mRNAs until needed. We refer to these granules as ‘SG-like’ given the apparent absence of stress, yet axotomy triggers partial disassembly of these axonal granules with the release of mRNAs for translation in axons to support regenerative growth [91,92,93]. Similarly, fragile X messenger ribonucleoprotein 1 (FMRP) functions as an RNA-binding protein to regulate RNA localization, but it can also be detected in SGs and regulates localized mRNA translation [94]. KHSRP (also called ZBP2 and MARTA1) exhibits multifunctionality, acting as an RNA-splicing factor in the nucleus, but it is also needed to localize some mRNAs and has functions in mRNA decay promotion [95]. This multifunctionality is a prevalent characteristic of RBPs, with different RNA-binding domains and protein–protein interaction domains driving these different functions [4].

Many RNPs go through a biophysical process of liquid–liquid phase separation (LLPS) to form granules that function as membrane-less, organelle-like structures within the cytoplasm [96]. This ability is imparted by intrinsically disordered regions (IDRs, also termed ‘low-complexity domains’), which are quite common in RBPs. Indeed, approximately 70% of human RBPs are reported to have at least one IDR extending over a 30-residue length [97,98]. IDRs provide a substrate for both intra- and intermolecular interactions that can modulate LLPS. For example, G3BP1 has three IDRs, with IDR 1 and 3 interactions providing a means of blocking LLPS. RNA binding to G3BP1 disrupts this intramolecular interaction and decreases G3BP1’s threshold for LLPS [99]. Phosphorylation of G3BP1 on Serine 149 (S149) within IDR 1 by Casein Kinase 2α (CK2α) following PNS nerve injury increases the protein’s threshold for LLPS and decreases its affinity for RNA interactions [92,99]. Interestingly, CK2α is introduced into injured axons through an axonal protein synthesis cascade that spatially and temporally restricts axonal CK2α activity to determine when and where G3BP1 is phosphorylated [92]. In non-neural systems, human G3BP1’s LLPS threshold is also elevated by Lysine 376 acetylation of the protein’s RNA-binding domain, which lies within IDR 3. Acetylation decreases G3BP1’s RNA-binding affinity and triggers SG disassembly [100]. It is not clear if this occurs in neurons or whether phosphorylation and acetylation could have synergistic or additive effects on G3BP1 LLPS and mRNA translation. Although axonal G3BP1 granules contain many of the same constituents as traditional SGs, it is not clear if these granules are in fact true SGs given that they exist in the apparent absence of stress.

Although these SG-like granules in axons contain many proteins in canonical SGs, their persistent presence in unstressed axons raises the question of whether the distal axonal environment itself imposes a form of intrinsic stress. This idea has gained traction in light of the pathology of neurodegenerative diseases. Phosphorylated Tar-DNA binding protein 43 (TDP-43), for example, forms pathological axonal aggregates in amyotrophic lateral sclerosis (ALS), including G3BP1 [101]. Pathological mutants of Fused in Sarcoma (FUS) and T-cell Intracellular Antigen 1 (TIA1) also form aggregates in the neuronal cytoplasm in ALS [102]. Interestingly, some neurological disease-linked proteins have been shown to bind to RNAs, with some undergoing LLPS-like transitions, including TAU (Alzheimer’s disease), HTT (Huntington disease), and LIS1 (Lissencephaly and related brain malformations) [103,104,105,106]

Based on our targeted mass spectrometry (MS) and RNA affinity mass spectrometry (RAMS) studies using extruded PNS nerve axoplasm isolates, we estimate that axons contain well over 100 RBPs. The axoplasm isolates are prepared under detergent-free conditions [107], so RBPs that are closely associated with cytoskeletal or transmembrane proteins can be missed or underrepresented. Thus, it is worth mentioning that absence in these MS studies does not equate to a lack of presence in the axonal compartment. For example, ZBP1 was not detected in RAMS or spectral-targeted MS detections [47,77], but ZBP1 clearly is present in axons, as determined by immunolocalization and functional analyses for *Actb* mRNA’s localization motif [108,109]. Nonetheless, some generalizations can be drawn from our current knowledge of RBP and mRNA motif interactomes. RAMS studies using synthetic RNAs corresponding to functionally characterized axonal localization motifs clearly show that multiple RBPs can bind to a single motif. These may be RBP complexes or individual RBPs serving different functions, and proteins presumed to spend most of their time in the nucleus do indeed contribute to RNA dynamics in axons [76,77,110]. Furthermore, affinity isolation of individual axonal RBPs from PNS axoplasm shows that they bind to 10s to 100s of different mRNAs [76,77], supporting the concept of RNA regulons and underscoring the complexity of RNPs as key regulatory platforms in axons.

### 2.5. Translational Regulation of Axonal mRNAs

The translation of mRNAs in axons is regulated as in cell compartments and cell types by translation factors that determine the initiation and elongation of mRNAs [4]; this includes both cap-dependent and cap-independent (internal ribosome entry site [IRES]) translation [65]. In addition, extracellular stimuli and the axonal physiology can determine when and where an mRNA is translated (Table 2). This fits with the notion of an ‘on demand’ protein supply as a central reason for the local synthesis of axonal proteins, circumventing delays imposed by long-distance transport from the soma [111]. Axon pathfinding stimuli can modulate the translation of different mRNAs in axons depending on whether the stimulus is attractive or repulsive [21]. Consistent with this, ribosome profiling of optic nerve axons has shown that the axonal translatome changes at different stages of axon growth—i.e., the population of ribosome-bound mRNA in a growing axon is distinct from when the axon reaching its target undergoes synaptogenesis [26].

In mature PNS axons, axotomy triggers rapid and localized translation of stored mRNAs. Axonal proteins synthesized in the early stages after injury are used for retrograde signaling and are locally used to initiate growth cone formation to support regeneration [3]. Calcium entry from the extracellular environment into the injured axon is a key event for initiating axotomy-dependent axonal mRNA translation. Translation of a number of mRNAs, including *Kpnb1*, *Ranbp1*, *Stat3α*, *mTor*, *Calr*, *Bip* (*Grp78*), *Creb3* (*Luman*) and *Khsrp*, are specifically increased by elevated axoplasmic calcium [20,40,42,47,48,50,53,92]. Axotomy also activates an intrinsic stress response (also termed ‘unfolded protein response’) locally in the axons [50,112]. This response precipitates from a decrease in nascent protein folding activity within the ER and results in phosphorylation of the translation factor eIF2α, which blocks general protein synthesis but paradoxically upregulates the translation of mRNAs whose proteins are needed for response to cell stress [113]. Conditions that lead to eIF2α Serine 51 phosphorylation in axons have been shown to activate the translation of *Creb3*, *Calr*, *Bip*, *Kpnb1*, *mTor*, and *Khsrp* mRNAs [43,47,50,92]. Since eIF2α^PS51^ blocks the translation of most mRNAs, the elevated axoplasmic calcium that leads to eIF2α phosphorylation through PERK needs to decline to near pre-injury levels for the translation of *Csnk2a1* and other growth-associated mRNAs [92]. *Csnk2a1* mRNA encodes CK2α, which phosphorylates G3BP1 to increase axonal mRNA translation through G3BP1 granule disassembly [92]. Responses to increased eIF2α^PS51^ can also be modified in terms of which mRNAs are translated based on the eIF2Bε level and phosphorylation status [114]. Phosphorylation of other translation factors and their activity-modulating proteins (e.g., eIF4E, 4EBP1/2) as well as ribosomal proteins (e.g., RPS6) through activation of signaling cascades (e.g., PI3K→mTOR) in axons can increase local protein synthesis [115].

Environmental stimuli from the growth-permissive environment of the PNS presumably further increase the translation of growth-associated mRNAs, since neurotrophic factors have been shown to increase the translation of axonal *Gap43*, *Actb*, and *Prenyl-Cdc42* mRNAs {Yoo, 2013 #6337; [73]}. However, not all mRNAs translated in PNS axons are growth-promoting. Axonally synthesized KHSRP slows axon growth by promoting localized decay of axon growth-associated mRNAs, with genetic deletion of KHSRP accelerating PNS nerve regeneration [47]. From a pathological context, Alzheimer’s disease-linked amyloid β (Aβ) peptide has also been shown to stimulate translation of axonal *Atf4* mRNA through a local intrinsic stress response that is postulated to lead to neurodegeneration [49]. Taken together, the specificity at which mRNAs are translated when in axons determines directed growth, response to injury, and impact on survival.

**Table 2:** Stimuli and mechanisms regulating axonal mRNA translation.

Stimulus	Effects on Translation	Target mRNA Examples	Mechanism and Outcome(s) [References]
Axotomy	Rapid Ca^2+^-dependent translation activation	*Kpnb1*, *Calr*, *Luman/Creb3*, *mTor*	Initiates retrograde signaling, supports growth cone formation, promotes further axonal mRNA translation [42,43,51,116]
mTOR activation	Stimulates CAP-dependent protein synthesis under normo-calcemic conditions	*Csnk2a1*, *Nrn1*	Phosphorylates eIF4E, 4EBP1/2, and RP S6 [42,92,117]
Axon pathfinding/guidance cues	Receptor-based signaling	*Rhoa*, *Actb*, *Cofilin*	Signaling cascades converge on translation factors, with sequestration of mRNAs and translational machinery near receptors [118,119,120,121,122]
Neurotransmitter (Glutamate)	Unknown, presumably ionic alterations	unknown	[123]
Amyloid β peptide	Converges on intrinsic stress response (ISR) pathway for eIF2α phosphorylation	*Atf4*	Induces neurodegeneration linked to Alzheimer’s disease [49]

### 2.6. Approaches to Modifying the Axonal Translatome to Increase Axon Regeneration

**Figure 1:**
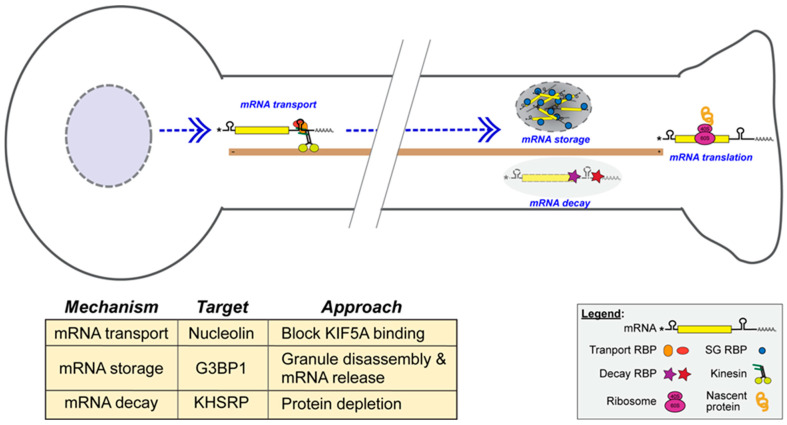
Leveraging localized translation for developing neural repair therapeutics. Schematic of a neuron illustrating the key molecular determinants that regulate the specificity, timing, and levels of protein synthesis from localized mRNAs. These determinants include mRNA transport and stability, granule dynamics, ribosome recruitment, and translation initiation. Therapeutic strategies that target these mechanisms to enhance axon regeneration are summarized in the accompanying table, along with their corresponding molecular targets and approaches. For example, the DNA aptamer AS1411 disrupts the interaction between nucleolin and the kinesin motor protein KIF5A, impairing axonal mRNA transport and offering a targeted means of modulating transcript localization. A G3BP1-derived peptide promotes stress granule disassembly by lowering the threshold for liquid–liquid phase separation, thereby enhancing local translation. Additionally, targeting the RNA-binding protein KHSRP through protein depletion reduces its promotion of mRNA decay, stabilizing growth-associated transcripts and enhancing their translation in regenerating axons. Together, these strategies exemplify how manipulation of localized translational pathways can be harnessed to promote neuronal repair.

Considering the contributions of axon-synthesized proteins to the axon regeneration, transport, storage, and stability of axonal mRNAs, which together modulate axonal protein synthesis, they are appealing targets for exogenously increasing axon regeneration (Figure 1). One well-characterized example is the axonal translation of *Kpnb1* mRNA, which encodes Importin-β1, a retrograde signaling protein involved in the initiation of transcriptional responses to injury. While Importin-β1 supports regeneration-associated gene expression, this protein has also been linked to cell-size sensing, and the depletion of axonal *Kpnb1* mRNA can similarly increase axon growth rates [110,116]. *Kpnb1* mRNA requires nucleolin (NCL) protein for its axonal localization, and decreasing NCL-mediated anterograde transport has been shown to increase rates of axon growth [110,124]. This has been achieved with a DNA aptamer, AS1411, which blocks NCL’s interaction with the KIF5A motor protein attenuating anterograde or retrograde axonal transport (Figure 1) [110,125]. Anterograde transport of the *Kpnb1* mRNA-NCL complex and retrograde transport of Importin-β1 plus cargo proteins allows the neuron to sense its axon length for gauging materials needed for growth [126]. Axonal mRNA storage in G3BP1 granules has also provided a target for accelerating axon regeneration. A cell-permeable peptide from G3BP1’s IDR 1 (amino acids 190–208) causes axonal G3BP1 granule disassembly, increases axonal mRNA translation, accelerates PNS axon growth, and promotes CNS axon growth (Figure 1) [91,93]. The mechanisms underlying this granule disassembly remain unknown. RNA stability can also be regulated in axons, with KHSRP decreasing the axonal levels of *Gap43*, *Snap25*, and *Prenyl-Cdc42* mRNAs and slowing axon growth (Figure 1) [47,73]. Though *Khsrp* mRNA translation is increased by elevated calcium in axons, its protein levels remain elevated throughout regeneration in rodent PNS axons [47]. Finding a means of blocking this axonal KHSRP elevation could provide an effective means of promoting nerve regeneration that may also be applicable to the CNS.

Taken together, these examples demonstrate that molecular interventions targeting axonal RNA localization, storage, and stability can effectively modulate the axonal translatome to promote regeneration. Developing therapeutics that selectively manipulate these regulatory steps may unlock new strategies to enhance repair in both the peripheral and central nervous system.

## 3. Summary and Perspectives

Axons utilize localized protein synthesis for axon growth, axon-to-soma signaling, and sustaining axon function. The distances that axons extend both provide a unique environment requiring localized protein synthesis and an advantageous experimental system for delineating the mechanisms modulating this subcellular post-transcriptional regulation. The availability of high-sensitivity detection of RNAs and proteins has fueled remarkable advances for identifying which proteins are made locally in axons. Complementary mechanistic studies have shed light on how axonal mRNA transport, translation, and stabilization/destabilization regulate the axon’s proteome. In the PNS, injury-induced activation of local translation is essential for initiating regeneration. The requirement for axonally synthesized proteins in the early stages of regeneration highlights several promising therapeutic targets to accelerate functional recovery.

Importantly, localized mRNA translation also occurs in CNS axon regeneration, and mounting evidence suggests that developing tools to manipulate mRNA transport, translation, and survival holds promise for promoting CNS axon regeneration, where axonal repair is typically limited [91]. Alterations in axonal mRNA transport and translation have been reported in CNS and PNS neurodegenerative disorders, including ALS and chemotherapy-induced neuropathy [44,101,127,128]. Price and Inyang (2015) showed similarities between the translation control mechanisms involved in neuropathic pain and memory/synaptic plasticity [129]. Axon injury shares some similarities with the initiation of an unfolded protein response after axotomy. It is intriguing to speculate that axons and dendrites use common mechanisms for different outcomes by altering the magnitude of the response and the specific mRNAs translated by those stimuli. Together, these findings emphasize the importance of understanding compartment-specific RNA regulation and highlight new opportunities to therapeutically manipulate local translation as a means of promoting regeneration and neural repair.
